# Hydrothermally Grown Globosa-like TiO_2_ Nanostructures for Effective Photocatalytic Dye Degradation and LPG Sensing

**DOI:** 10.3390/molecules29174063

**Published:** 2024-08-27

**Authors:** Mutcha Shanmukha Rao, Benadict Rakesh, Gunendra Prasad Ojha, Ramasamy Sakthivel, Bishweshwar Pant, Kamatchi Jothiramalingam Sankaran

**Affiliations:** 1 CSIR-Institute of Minerals and Materials Technology, Bhubaneswar 751013, India; shanmukha.2021@immt.res.in (M.S.R.); rsakthivel@immt.res.in (R.S.); kjsankaran@immt.res.in (K.J.S.); 2 Academy of Scientific and Innovative Research (AcSIR), Ghaziabad 201002, India; 3 Carbon Composite Energy Nanomaterials Research Center, Woosuk University, Wanju 55338, Republic of Korea; gpojha10@gmail.com

**Keywords:** titanium dioxide, hydrothermal method, nanostructures, photocatalytic dye degradation, gas sensor

## Abstract

The rapid expansion of industrial activities has resulted in severe environmental pollution manifested by organic dyes discharged from the food, textile, and leather industries, as well as hazardous gas emissions from various industrial processes. Titanium dioxide (TiO_2_)-nanostructured materials have emerged as promising candidates for effective photocatalytic dye degradation and gas sensing applications owing to their unique physicochemical properties. This study investigates the development of a photocatalyst and a liquefied petroleum gas (LPG) sensor using hydrothermally synthesized globosa-like TiO_2_ nanostructures (GTNs). The synthesized GTNs are then evaluated to photocatalytically degrade methylene blue dye, resulting in an outstanding photocatalytic activity of 91% degradation within 160 min under UV light irradiation. Furthermore, these nanostructures are utilized to sense liquefied petroleum gas, which attains a superior sensitivity of 7.3% with high response and recovery times and good reproducibility. This facile and cost-effective hydrothermal method of fabricating TiO_2_ nanostructures opens a new avenue in photocatalytic dye degradation and gas sensing applications.

## 1. Introduction

Environmental pollution, water scarcity, and wastewater management pose a critical convergence of threats to both human and ecological health. Exponential population growth and rapid industrial development have led to the release of organic pollutants such as methylene blue, methyl orange, congo red, rhodamine B, and crystal violet dyes into water bodies, thereby disrupting aquatic ecosystems [1]. Additionally, industrial activities, wildfires, and volcanic eruptions introduce a diverse range of contaminants into the environment [2]. The subsequent dissemination of noxious gases resulting from these activities significantly impacts living organisms, posing considerable health risks [3]. Metal oxides, particularly TiO_2_, ZnO, WO_3_, In_2_O_3_, SnO_2_, and Fe_2_O_3_ have dominated photocatalysis and gas sensing applications due to their abundance, affordability, and ability to tailor their properties for specific needs [4,5]. Among the various metal oxide nanostructured materials, TiO_2_ nanostructures constitute a versatile material with significant potential in environmental remediation, clean energy generation, and consumer products. The wide band gap and potent oxidation power of TiO_2_, when activated by ultraviolet (UV) or visible light irradiation, demonstrates significant promise for addressing environmental challenges through its photocatalytic and gas sensing capabilities [6,7,8]. However, TiO_2_ films face challenges such as slow response and recovery times, low sensitivity, poor selectivity, and the need for high operating temperatures. This understanding has spurred the development of nanostructured TiO_2_ with diverse morphologies (nanoflowers, nanowires, nanosheets, nanorods, etc.).

Nanostructured TiO_2_ photocatalysts have garnered considerable attention due to their unique morphologies and properties [9]. These nanostructures possess a significantly increased surface area compared to their bulk counterparts, providing abundant active sites for light absorption and reactant interaction. Since its pioneering discovery by Fujishima and Honda in the early 1970s, the photocatalytic activity of TiO_2_ has undergone significant advancements [10,11]. H. Almohamadi et al. [9] reported that the efficacy of the TiO_2_ photocatalyst is also intrinsically linked to its structural characteristics. Hao et al. [12] reported the effect of overlayered TiO_2_ and underlayer CuO on film morphology and the photocatalytic activity of the nanocomposite materials. Montero et al. [13] reported that the surface micro-nanostructuring of TiO_2_ films, studying the photocatalytic properties of formic acid degradation under UV light. On the other hand, environmental concerns are driving the development of advanced gas sensing devices to detect hazardous gases like LPG, H_2_S, CO, and NH_3_. These devices play a vital role in safety, pollution monitoring, and industrial control. Recently, it is reported that TiO_2_ nanostructures have shown significant potential in detecting a variety of gases, including NO_x_, CO, H_2_S, NH_3_, acetone, ethanol, CH_4_, and H_2_ [14], because of their enhanced surface area and high sensitivity [15]. 

Despite extensive research on TiO_2_ for photocatalysis and gas sensing applications, the primary challenge is in the fabrication of these TiO_2_ nanostructures. TiO_2_ nanostructures can be synthesized using methods such as hydrothermal synthesis, chemical vapor deposition, sol–gel, pulse laser deposition, electrodeposition, RF sputtering, and DC sputtering [7,16,17]. Particularly, hydrothermal synthesis offers a versatile and efficient method for creating TiO_2_ nanostructures [18]. This technique allows for precise control over the material’s properties, making it valuable for developing advanced materials with diverse applications [19]. A key advantage lies in its ability to tune crystallinity and morphology by adjusting reaction parameters like temperature, pressure, and duration [20]. Notably, the autoclave’s pressure increases alongside temperature, exceeding the boiling point of water [19].

Herein, this work demonstrates a cost-effective fabrication of hydrothermally synthesized TiO_2_ nanostructures that exhibit promising photocatalytic activity and LPG sensing capabilities. The well-defined globose flower-like TiO_2_ nanostructures (GTNs), having a high ratio of oxidative-to-reductive surface sites, promote efficient oxidation and reduction reactions during photocatalysis and gas sensing, respectively. The mechanism of the formation of globosa flower-like TiO_2_ nanostructures, along with the enhanced photocatalytic dye degradation as well as superior LPG sensing characteristics are explored.

## 2. Results and Discussion

### 2.1. Materials Characteristics

**Figure 1:**
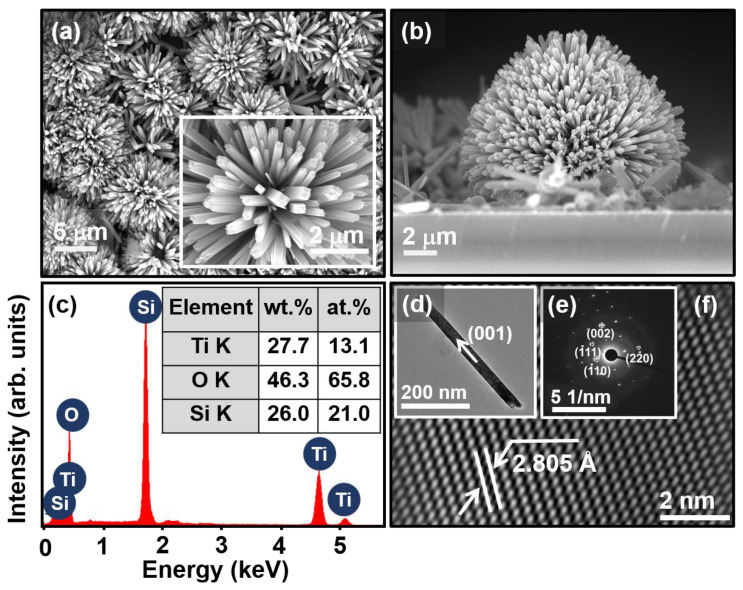
(**a**) Plain-view FESEM image of GTNs. (**b**) Cross-sectional FESEM image of GTNs. (**c**) FESM–EDX spectrum of GTNs. (**d**) TEM micrograph with corresponding SAED pattern of (**e**) GTNs synthesized using hydrothermal method. (**f**) HRTEM micrograph of the selected lattice fringes area of GTNs.

The surface morphology of GTNs was examined using the plain-view FESEM. Figure 1a reveals the formation of uniformly distributed globosa flower-like nanostructures of TiO_2_ on glass substrate. A well-aligned configuration of GTNs refers to an arrangement where the individual nanorods, which resemble flower petals, exhibit a preferred orientation relative to a specific axis. The magnified plain-view FESEM image shown in the inset of Figure 1a represents the nanopetals of GTNs with a diameter of ~250 nm and length of around 2.1 μm. The height of the GTNs was 8.4 μm, which was determined from the cross-sectional view FESEM micrograph shown in Figure 1b. The FESEM–EDX spectrum shown in Figure 1c confirms the presence of Ti, O, and Si elements in the GTNs. The specific surface area (SSA) of GTNs was determined (using ImageJ software-1.54j version) to be up to 0.921 m^2^/g [21]. The surface area analysis was conducted on GTNs by using the FESEM plot profiles. (Detailed geometrical calculations of SSA are provided in the Supporting Information). Figure 1c shows the intense Si line corresponding to the underlying glass substrate, upon which the GTNs were grown. EDX can provide valuable qualitative and semi-quantitative information about the elemental composition of GTNs. Improving the EDX quantification of GTNs depends on several factors such as sample preparation, detector efficiency, and optimizing the beam energy [22]. At high temperatures and autogenous pressure under hydrothermal conditions, oxygen from the TiO_2_ film can diffuse into the glass substrate, creating an oxygen-rich interface [23]. Figure 1c shows the obtained wt.% and at.% of EDX data corresponding to Ti, O, and Si. The TiO_2_ nanorod was oriented along the [001] crystallographic direction and projected on the [110] zone axis (Figure 1d), which is in good agreement with the FESEM micrographs (Figure 1a,b). Figure 1e displays the selective area electron diffraction (SAED) pattern of GTNs, revealing the single-crystalline nature of the material. The SAED pattern confirms the growth of tetragonal rutile GTNs. In this study, the predominant crystallographic planes of the tetragonal rutile GTN structure are identified as $\left(\bar{1}10\right)$, $\left(\bar{1}11\right)$, (002), and $\left(2\bar{2}0\right)$, respectively. Moreover, Figure 1f displays the HRTEM micrograph of the GTNs. The measured *d*-spacing value between the lattice planes is 2.805 Å that corresponds to the (111) lattice plane of TiO_2_.

**Figure 2:**
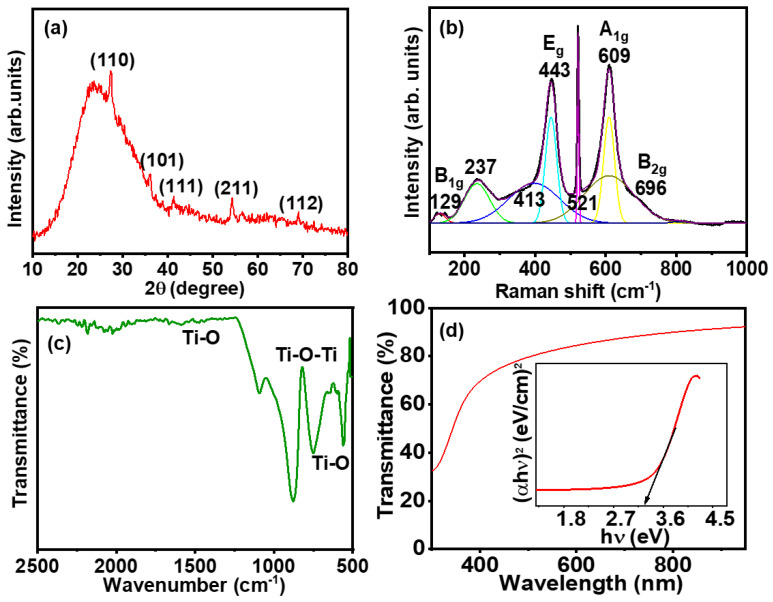
(**a**) XRD pattern, (**b**) Raman spectrum, (**c**) FTIR spectrum, and (**d**) UV-vis transmittance spectrum of GTNs. The inset of (**d**) shows the estimation of optical bandgap of GTNs.

As shown in Figure 2a, the XRD pattern of the GTNs shows peaks at Bragg diffraction angles (2θ) of 27.4°, 36.0°, 41.3°, 54.4°, and 69.0°, corresponding to the Miller indices of the (110), (101), (111), (211), and (112) planes, respectively, which are characteristic of the rutile phase. GTNs in the tetragonal phase (JCPDS: 01-076-0321, space group: P4_2_/mnm) had unit cell parameters a = b = 4.625 Å, c = 2.9878 Å. The investigation of GTNs using XRD analysis, along with FESEM–EDX and HRTEM, confirms the formation of rutile GTNs.

Raman scattering was employed on the GTNs to identify the vibrational modes of the material [24]. Figure 2b exhibited the rutile phase of TiO_2_, corroborating the findings obtained from the XRD analysis. The typical Raman active modes of anatase TiO_2_ are A_1g_, 2B_1g_, and 3_Eg_, while the Raman active modes for rutile TiO_2_ films are A_1g_, B_1g_, B_2g_, and E_g_ [25,26]_._ The Raman spectrum of GTNs exhibits prominent bands at higher frequencies, specifically at 129 cm^−1^, 443 cm^−1^, and 609 cm^−1^, which correspond to the B_1g_, E_g_, and A_1g_ vibrational modes of rutile TiO_2_, respectively. Additionally, minor peaks are observed at 237 cm^−1^, 413 cm^−1^, 521 cm^−1^, and 696 cm^−1^, which correspond to B_2g_ vibrational modes of rutile TiO_2_. A_1g_ and B_1g_ represent in-plane modes, while the E_g_ mode corresponds to the out-of-plane mode. The E_g_ mode involves the symmetric stretching of O–Ti–O bonds and exhibits a second-order scattering feature, with the most notable peak observed at 237 cm^−1^. The A_1g_ and E_g_ modes signify the vibrational characteristics of the crystal lattice, indicative of the high crystallinity nature of the GTNs [27].

Figure 2c shows that the bands in the range 4000 cm^−1^ to 400 cm^−1^ were attributed to strong absorption and vibrational bands of GTNs. The distinct broad band peaks at 565 cm^−1^, 730 cm^−1^, and 882 cm^−1^ were assigned to the low-frequency bending vibrations of Ti–O and Ti–O–Ti (absorption bands). The presence of a Ti–O bond at peak at 1523 cm^−^¹ confirms the formation of TiO_2_ [28]. The inset of Figure 2d shows the transmittance (%) of GTNs that was calculated in the wavelength range of 300–1000 nm. The optical transmission in the UV–vis–near-infrared region is 70–90%. Figure 2d shows the UV–vis–near-infrared spectrophotometer of the GTNs. The estimated optical bandgap of the GTNs evaluated from Figure 2d using a Tauc plot [29] is 3.06 eV.

It is clearly revealed from the materials characteristics that there is a formation of well-defined single-crystalline rutile phase GTNs. However, the underlying growth mechanism for GTNs remains elusive. Basically, the formation of metal oxide nanostructures follows a rapid nucleation of small particles at the initial stage, followed by their aggregation. Subsequently, these smaller particles dissolve while larger ones grow due to Ostwald ripening and lead to the formation of nanostructures [30,31]. Moreover, Yang et al. [32] proposed that the anisotropic growth of rutile TiO_2_ nanoflower arrays is governed by the atomic configuration at the nucleation sites on substrates or within precursors. It is also suggested that the analogous titanium atomic arrangement between metallic titanium and rutile TiO_2_ facilitates the vertical growth of rutile TiO_2_ from the (001) plane of the TiO_2_ film [32,33,34]. On the other hand, Ali et al. [35] described the hydrolysis of the TiO_2_ precursor and the condensation process to advance through a balance of two key processes: olation and oxolation. The growth of highly crystalline TiO_2_ nanorods is significantly influenced by process parameters such as precursor concentration, reaction duration, temperature, and subsequent annealing time [34,35].

**Figure 3:**
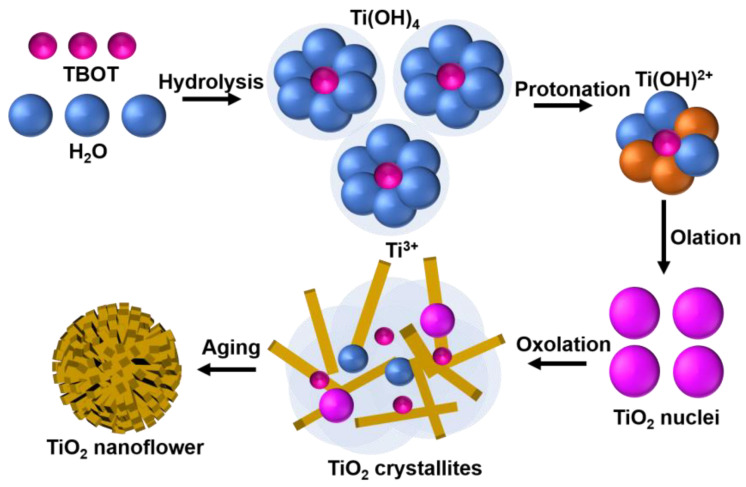
Schematic illustrations on the growth mechanism of GTNs.

Building upon the knowledge from earlier findings, the fabrication mechanism of hydrothermally synthesized GTNs is derived (Figure 3). During the hydrolysis process, titanium (IV) butoxide (TBOT) precursor reacts with water (H_2_O). The coordination number of Ti^4+^ increases from +4 to +6 using its vacant d-orbitals to accept oxygen lone pairs forming Ti–O bonds [33,36]. Hydrolysis of the titanium precursor occurs, resulting in the exchange of butyl groups with hydroxyl (OH) groups [37]. The liberated protons (H⁺) from the acid solution further react with the hydrolysed precursor, leading to the formation of the titanium hydroxide intermediate, TiOH^2+^ [19]. The protonated titanium hydroxide intermediates can readily condense through the interaction with hydroxyl groups (OH^−^) on neighbouring TiO₆ octahedra. Subsequent dehydration (olation) steps, driven by the removal of water molecules (H_2_O), lead to the formation of the TiO_2_ nuclei. Notably, chloride ions (Cl^−^) from the acidic solution play a crucial role in deprotonation (oxolation) processes, influencing the growth of the GTNs [38].

Hydrolysis: Ti(OR)_4_ + 4H_2_O → Ti(OH)_4_ + 4ROH (1)

Dehydration: Ti(OH)_4_ + Ti(OH)_4_ → 2TiO_2_ + 4H_2_O (2)

The entire reaction sequences for the formation of GTNs via the hydrothermal process are as follows:

Ti(OH)_4_ + Ti(OR)_4_ → 2TiO_2_ + 4ROH (3)

Ti(OR)_4_ + 2H_2_O → TiO_2_ + 4ROH (4)

GTNs exhibit well-defined rutile TiO_2_ nanostructures having a high surface area and abundant active sites, which promotes efficient oxidation and reduction reactions during photocatalytic dye degradation and gas sensing applications.

### 2.2. Photocatalytic Degradation of MB Dye

**Figure 4:**
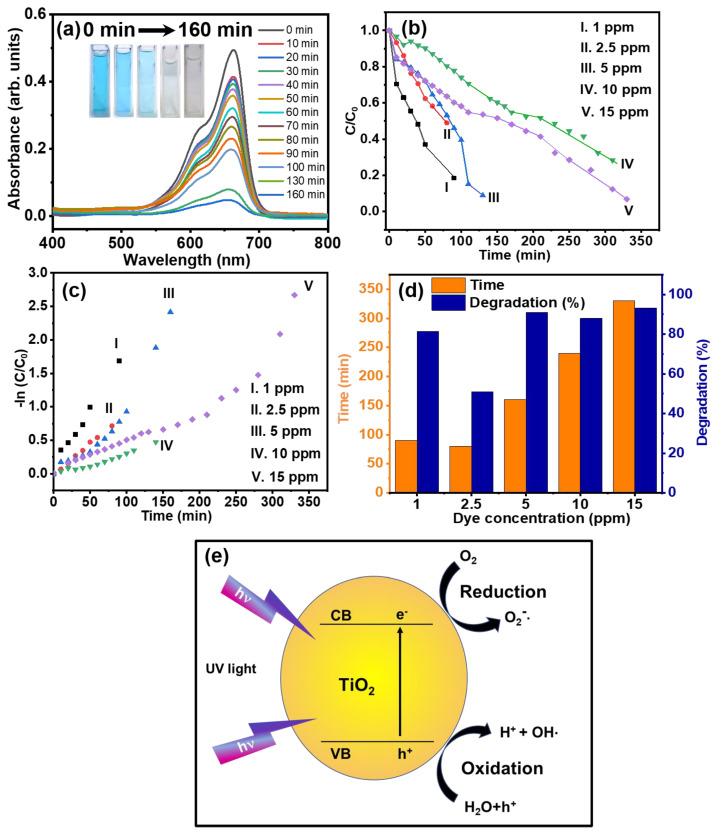
(**a**) UV-vis absorbance spectrum of MB (5 ppm), (**b**) photocatalytic degradation of MB (1 ppm, 2.5 ppm, 5 ppm, 10 ppm, and 15 ppm) under UV irradiation, (**c**) the rate constants for MB dye degradation, (**d**) the photocatalytic degradation (%) of MB dye at various concentrations, and (**e**) the photocatalytic dye degradation mechanism of GTNs.

Figure 4a presents the investigation of the photocatalytic activity of rutile GTNs using the photodegradation of MB dye under UV light irradiation. Absorbance spectra shown in Figure 4a reveal that the GTNs exhibit excellent degradation performance with a 91% degradation of 5 ppm of MB dye within 160 min. The inset of Figure 4a displays the images of colour change during MB dye degradation. The photocatalytic degradation efficiency of GTNs was investigated at varying initial MB concentrations (1 ppm, 2.5 ppm, 5 ppm, 10 ppm, and 15 ppm) as shown in Figure 4b. The plot shows the normalized concentration (C/C_o_) of MB versus irradiation time for GTNs with varying initial dye concentrations. Notably, 5 ppm of MB resulted in the highest degradation efficiency compared to other initial MB concentrations. Furthermore, the degradation rate follows a pseudo-first-order kinetic model, where the rate constant can be determined by [39] the following:

ln (C/C_o_) = −Kt (5)

where C is the degraded concentration, C_o_ is the initial concentration, K is the rate constant of the reaction, and time (t) is the degradation time.

The rate constants (k) for MB degradation were determined at various concentrations of dye as shown in Figure 4c. The rate constants followed a decreasing trend: k(MB) = 0.017 min^−1^ (1 ppm) > 0.009 min^−1^ (2.5 ppm) > 0.006 min^−1^ (5 ppm) > 0.004 min^−1^ (10 ppm) > 0.002 min^−1^ (15 ppm). The efficiency of MB dye degradation is determined by the following equation:

R = (C_o_ − C)/C_o_ × 100% (6)

Figure 4d shows the degradation percentages of MB dye at various concentrations. The degradation achieved was 81% for 1 ppm in 90 min, 51% for 2.5 ppm in 80 min, 91% for 5 ppm in 160 min, 87% for 10 ppm in 240 min, and 71% for 15 ppm in 250 min, respectively. The highest degradation efficiency (91%) was achieved for 5 ppm MB within a relatively short irradiation time (160 min). The combined data from Figure 4c,d demonstrate that the photocatalytic degradation efficiency of MB is directly influenced by the concentration. Lower MB concentrations facilitate faster and more efficient degradation, while higher concentrations require longer irradiation times and achieve lower degradation efficiencies. This is because upon absorbing a photon with enough energy, an electron in the valence band of TiO_2_ is excited to the conduction band. To elucidate the reasons behind the differences in photocatalytic activities, we must delve into the fundamental mechanism of semiconductor photocatalysis. Identifying the most effective nanomaterials for dye degradation requires a thorough evaluation of various parameters and the specific properties of the target dyes. Factors like composition, morphology, surface area, and crystallinity of nanomaterials play a crucial role in determining their degradation rate. Table 1. is provided to facilitate a clearer understanding of the photocatalytic MB dye degradation efficiency analysis across various nanomaterials (Such as MoO_3_/TiO_2_/5%rGO, Fe_2_O_3_/TiO_2_, Cu-CdS, Ag doped ZnO, ZnO-CNT, SnO_2_ nanotubes, and different TiO_2_ nanostructures) reveals distinct performance variations [40,41,42,43,44,45,46,47,48].

Packialakshmi et al. [49] demonstrated that the metal oxide nanocomposites (ZnO/SnO_2_/rGO) displayed an enhanced photocatalytic dye degradation due to the synergistic interactions between the nanocomposites, resulting in improved charge separation and reduced recombination. Figure 4e shows a schematic representation of the photocatalytic mechanism of GTNs; the photocatalytic activity of GTNs indeed arises from the absorption of ultraviolet (UV) light below 387 nm, which corresponds to a band gap of 3.06 eV (cf. Figure 2d) [50,51]. Light absorption triggers the excitation of an electron (e^−^) from the valence band (VB) to the conduction band (CB), creating a corresponding hole (h⁺) in the VB. The photogenerated holes (h⁺) possess strong oxidizing power and can react with surface-adsorbed water molecules (H_2_O) and other pollutant species. This creates an electron–hole pair that can participate in redox reactions on the surface of the GTNs, leading to various photocatalytic activities [52]. The photocatalytic process initiates the generation of hydroxyl radicals (•OH) and other highly oxidizing species (HOS), which effectively decompose and degrade organic pollutants into inert compounds, predominantly carbon dioxide (CO_2_) and water (H_2_O) [53]. The rate of electron–hole pair recombination depends on the light intensity and bandgap of the photocatalyst. The enhanced photocatalytic activity of GTNs can be attributed to their large surface area and the abundance of active sites. These features facilitate a greater extent of reactant molecule adsorption, leading to a higher number of reaction sites and promoting efficient photocatalysis.

### 2.3. LPG Sensing

**Figure 5:**
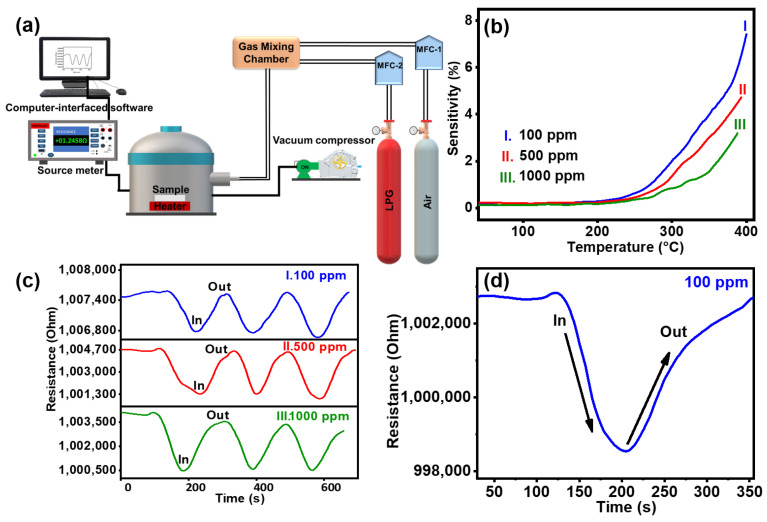
(**a**) Schematic of the LPG sensing instrument. (**b**) Sensitivity (%) of GTNs to LPG at different ppm levels. (**c**,**d**) Response time and recovery time of GTNs at 250 °C for concentrations of 100 ppm, 500 ppm, and 1000 ppm, respectively.

The rising popularity of LPG for cooking and industrial applications necessitates the development of reliable leak detection systems. LPG leakage causes accidents and affects the health of living beings due to it containing propane, butane, propylene, isobutane, and butylene. The accurate measurement of LPG leakage requires rapid and selective detection [54,55]. Aishwarya et al. [56] reported that the development of LPG sensors is paramount for public safety. The performance of LPG sensors can be improved using the synthesis method, fabrication technology, and dopant, catalyst, or advanced materials. The gas sensing mechanism relies on the adsorption and desorption of target gases on the surface of the sensing material. The GTNs hold significant potential as gas sensor materials owing to their high surface area, well-aligned architecture, and reduced agglomeration [57]. The LPG sensing performance of GTNs was systematically investigated by exposing them to LPG within a gas sensor unit. Figure 5a presents a schematic diagram of the gas sensing instrument, with GTNs employed as the sensing material and LPG used as the target gas. The target gas was diffused into the evacuated chamber at various concentrations (100 ppm, 500 ppm, and 1000 ppm). Experiments were conducted at different operational temperatures, including 40 °C, 100 °C, 200 °C, and 250 °C. As the operating temperature rises, increased thermal energy facilitates overcoming the activation energy barrier, leading to a linear augmentation in LPG response [54]. Figure 5b shows the sensing response (%) of the rutile GTNs at different ppm levels of LPG under various operating temperatures. At an operational temperature of 250 °C, the rutile GTNs exhibit a sensing response of 7.3%, 4.7%, and 3.1% to LPG concentrations of 100 ppm, 500 ppm, and 1000 ppm, respectively. The sensor demonstrates a pronounced enhancement in sensitivity as the concentration decreases. The diverse morphologies and distinct structure of the sensor offer ample and efficient gas diffusion channels along with active sites for gas molecule reactions [8]. The GTNs possess distinctive properties that enhance the adsorption and diffusion of target gas molecules both on its surface and within the interior regions of the TiO_2_ nanostructures [57]. Upon exposure to the target gas, the sensor consistently achieved excellent response and recovery values, demonstrating exceptional reproducibility. Figure 5c,d show that at an operational temperature of 250 °C, the response times of GTNs are 10 s, 11 s, and 12 s for concentrations of 100 ppm, 500 ppm, and 1000 ppm, respectively. Correspondingly, the recovery times are 80 s, 134 s, and 142 s for the same concentrations. The enhanced repeatability and long-term stability in detecting trace amounts of LPG are ensured by the high surface area and well-defined architecture of TiO_2_ nanostructures.

Tian et al. [8] reported that the gas sensing performance of TiO_2_ is strongly influenced by its controllable morphology. TiO_2_ nanostructures offer unique advantages such as increased surface area, faster electron transport, better gas permeability, and more active reaction sites. These factors collectively enhance TiO_2_ gas sensor capabilities. The LPG sensing mechanism of TiO_2_ is based on an oxidation and reduction reaction that occurs when the target gas interacts with the sensing material, and desorption of the gas molecules from the sensing material occurs when the temperature decreases [56,58]. When O_2_ molecules adsorb onto the surface of the TiO_2_ nanostructures, they extract electrons from the conduction band and subsequently trap them at the surface in the form of ions. The sensing mechanism of GTN sensors in air and under LPG exposure relies on the nature of the adsorbed oxygen ions, including O^2−^ (ads), O^−^ (ads), and O^2−^ (ads) on the film surfaces. These ionic species extract the electron from the conduction band [59]. The negative charge trapped in these oxygen species induces upward band bending, resulting in a substantial decrease in electrical resistance, consequently reducing the barrier height. The interaction between chemisorbed oxygen species and injected LPG molecules can be expressed as follows [2,54]:

C_n_H_2n+2_ + 2O^−^ → H_2_O + C_n_H_2n_-O + e^−^ (7)

where C_n_H_2n+2_ represents CH_4_, C_3_H_8_, and C_4_H_10_.

The reaction of these oxygen species with reducing gases, or the competitive adsorption and replacement of the adsorbed oxygen by other molecules, can decrease or even reverse the band bending. This results in a temporal change in the resistance of the TiO_2_ nanostructured sensor. GTNs exhibit exceptional sensing characteristics for various concentrations of LPG, including good reproducibility, fast response times, and quick recovery times. These advantageous properties are attributed to the high surface area and well-defined architecture of the TiO_2_ nanostructures, which offer numerous active sites for gas adsorption and interaction.

## 3. Materials and Methods

### 3.1. Materials

Hydrochloric acid (HCl) (Qualigens, Mumbai, India) (35.8–38.0 wt%), TBOT (Sigma-Aldrich, St. louis, MO, USA), glass substrates, and distilled water were utilized in the preparation of TiO_2_ nanostructures.

### 3.2. Hydrothermal Synthesis of GTNs

For the fabrication of GTNs, glass substrates with dimensions of 2 × 2 cm were employed. The substrates underwent a cleaning process involving ultrasonication in distilled water, acetone, and ethanol, each for a duration of 30 min. Prior to initiating the experiment, the hydrothermal autoclave reactor was thoroughly cleaned to prevent contamination. A total of 20 mL of HCl and 20 mL of deionized (DI) water, in a 1:1 ratio, was prepared. This solution was magnetically stirred at 500 rpm for 15 min at room temperature. Subsequently, 0.5 mL of TBOT was added dropwise over a period of 40 min, maintaining the room temperature throughout the process. The mixed solution was transferred into a Teflon liner (100 mL volume), and it was loaded in a hydrothermal autoclave reactor wherein the ultrasonically cleaned glass substrates were immersed. The deposition of GTNs was achieved by subjecting the autoclave to thermal treatment in a hot air oven at 150 °C for 12 h. This was followed by an aging process at ambient temperature for 24 h. The samples were rinsed with DI water and then films were dried on a hot plate at 60 °C for 15 min.

### 3.3. Characterization

The crystal structure of the GTNs was determined using X-ray diffraction (XRD; Rigaku Instrument, Model-Ultima IV, Tokyo, Japan, X-ray diffractometer) using Cu Kα radiation (λ = 1.54056 Å). The surface morphologies and elemental composition analysis of the samples were observed using field-emission scanning electron microscopy (FESEM; JSM-IT 800, JEOL, Tokyo, Japan) with an accelerated potential of 15 kV; energy-dispersive X-ray analysis (EDAX; ELECT SUPER, JSM IT300, Pleasanton, CA, USA); high-resolution transmission electron microscopy (HRTEM; JEM-F200 200 kV, JEOL, Tokyo, Japan); a Raman spectrometer (Renishaw Plc, Wotton-under-Edge, UK, inVia with a 532 nm laser); and a Fourier-transformed infrared spectrometer (FTIR, BRUKER ALPHA II, Billerica, MA, USA, ATR Mode) recorded in the 4000 cm^−1^ to 400 cm^−1^ was used to identify the functional groups. UV–visible transmittance of GTNs was recorded on a UV–vis–near-infrared spectrophotometer (UV-VIS-NIR; Agilent instrument, Cary 5000, Santa Clara, CA, USA) to estimate the band gap of GTNs.

### 3.4. Photocatalytic Dye Degradation of MB Dye

Photocatalytic activity of GTNs was examined by using MB (MB; C_16_H_18_ClN_3_S·xH_2_O) dye. A series of MB stock solutions with varying initial concentrations (1 mg/L, 2.5 mg/L, 5 mg/L, 10 mg/L, and 15 mg/L) were prepared via serial dilutions in double-distilled water for the photocatalytic dye degradation experiments. The degradation of MB was performed using various initial concentrations; aliquots of 4 mL each were prepared from MB solutions with concentrations of 1 ppm, 2.5 ppm, 5 ppm, 10 ppm, and 15 ppm. These were then introduced into a 100 mL quartz beaker containing a 10 mm × 10 mm catalyst within a fabricated reactor. A custom photocatalytic reactor was designed for the experiments, which consists of UV-C (Philips TUV 8 W G8T5) sources positioned at the top to irradiate the test solution placed in a beaker at the bottom. The distance between the UV light source and the test solution surface was maintained at a constant 10 cm. The photocatalyst and an aqueous dye solution were kept in the dark for 30 min to achieve equilibrium adsorption of the dye molecules onto the catalyst surface. The photocatalytic activity of hydrothermally prepared GTNs was evaluated under UV light irradiation. The concentration of MB was recorded at 663 nm using a UV–visible spectrometer (UV-1700, Shimadzu, Kyoto, Japan). Absorbance spectra were recorded at 10 min intervals to monitor dye degradation. These spectral measurements were utilized to evaluate the progression and degree of degradation.

### 3.5. LPG Sensing

LPG sensing experiments were carried out in a custom-made gas sensing chamber. The GTNs sample was positioned within a sealed chamber, and electrical contacts were created on the film surface using silver paste. The operating temperature varied from room temperature to 400 °C, monitored by a thermocouple. The samples were subjected to heating during the measurements. A pre-mixed gas stream containing LPG diluted in air at varying concentrations (ppm) was injected into the chamber using mass flow controllers. A source meter (Keithley 6430; Solon, OH, USA), interfaced with computer software, was employed to control the instrument. The LPG sensing characteristics and sensing responses were recorded by monitoring the changes in resistance with temperature variations at different LPG concentrations of 100 ppm, 500 ppm, and 1000 ppm. Sensitivity (%) was calculated using Equation (8), where resistance of air (R_a_) and resistance of gas (R_g_) under various conditions was calculated.

Sensitivity Response (%) = (R_air_ − R_gas_)/R_gas_ × 100 (8)

After the gas sensing experiment, the sample was heated at 400 °C for 30 min to desorb the species on the film surface, enhancing reproducibility.

## 4. Conclusions

Globosa-like TiO₂ nanostructures were synthesized using a simple and cost-effective hydrothermal method. Structural and compositional analyses confirmed the formation of single-crystalline rutile TiO₂ with characteristic Ti-O and Ti-O-Ti bonds with a direct optical bandgap of 3.06 eV. The growth mechanism on the formation of globosa-like TiO₂ nanostructures was derived. The photocatalytic degradation performance of the TiO₂ nanostructures was evaluated across a range of MB concentrations from 1 to 15 ppm. The nanostructures exhibited a significant MB dye degradation efficiency of approximately 91% within 160 minutes under UV light irradiation. Lower MB concentrations facilitated enhanced and accelerated degradation, whereas higher concentrations resulted in prolonged irradiation times and reduced degradation efficiency. Additionally, the nanostructures showed a 7.3% sensing response to 100 ppm LPG at 250°C, with rapid response and recovery times. The enhanced surface area and well-defined architecture synergistically contributed to attain remarkable photocatalytic and LPG sensing performance.

## Figures and Tables

**Table 1:** Comparison table on the photocatalytic dye degradation efficiency of GTN catalyst with other reported catalysts.

Catalysts	Light Source/Dye	Degradation Time	Dye Degradation (%)	References
MoO_3_/TiO_2_/5%rGO	UV light/MB	60 min	95%	[40]
Fe_2_O_3_/TiO_2_	UV light/MB	60 min	95%	[41]
Cu-CdS	Xe light/MB	260 min	89%	[42]
Ag doped ZnO	500 W halogen lamp/MB	200 min	95%	[43]
ZnO-CNT	UV light/MB	120 min	93%	[44]
SnO_2_ nanotubes	UV light/MB	180 min	80.2%	[45]
TiO_2_ nanorods	UV light/MB	330 min	96%	[46]
TiO_2_ decorated CNT	UV light/MB	180 min	85%	[47]
TiO_2_	UV light/MB	180 min	40%	[47]
Degussa p25	Solar light/MB	120 min	57%	[48]
Globosa like TiO_2_ nanostructures (GTNs)	UV light/MB	160 min	91%	Present work

## Data Availability

Data will be made available upon request.

## Supplementary Materials

The following supporting information can be downloaded at: https://www.mdpi.com/article/10.3390/molecules29174063/s1, Figure S1: (a) Plain-view FESEM image of GTNs. (b) Schematic representation of the FESEM plot profile, (c–e) different surface area plot analysis (ImageJ Software-1.54j version) of GTNs [60,61,62].
