# Supplementary material for: Hydrothermally Grown Globosa-like TiO2 Nanostructures for Effective Photocatalytic Dye Degradation and LPG Sensing
Source: Molecules. 2024 Aug 27;29(17):4063. doi: 10.3390/molecules29174063 (PMC11396606; doi:10.3390/molecules29174063)
Supplement: Supplementary file 1 [file molecules-29-04063-s001.zip › molecules-3143127-supplementary.pdf]

Supporting Information

Hydrothermally grown globosa-like TiO_2 nanostructures for effective photocatalytic dye degradation and LPG sensing

Mutcha Shanmukha Rao [1,2], Benadict Rakesh [1,2*], Gunendra Prasad Ojha [3], R. Sakthivel [1,2], Bishweshwar Pant [3,*] and K. J. Sankaran [1,2*]

[1] *CSIR-Institute of Minerals and Materials Technology, Bhubaneswar-751013, India; shanmukha.2021@immt.res.in (M.S.); benadict@immt.res.in (B.R.); kjsankaran@immt.res.in (K.J.)*

[2] *Academy of Scientific and Innovative Research (AcSIR), Ghaziabad-201002, India; rsakthivel@immt.res.in (R.S.)*

[3] *Carbon Composite Energy Nanomaterials Research Center, Woosuk University, Wanju, 55338, South Korea; bisup@woosuk.ac.kr (B. Pant); gpojha10@gmail.com (G. P OJha)*

*Correspondence: Kamatchi Jothiramalingam Sankaran; (kjsankaran@immt.res.in), Benadict Rakesh (benadict@immt.res.in,), B. Pant (bisup@woosuk.ac.kr)

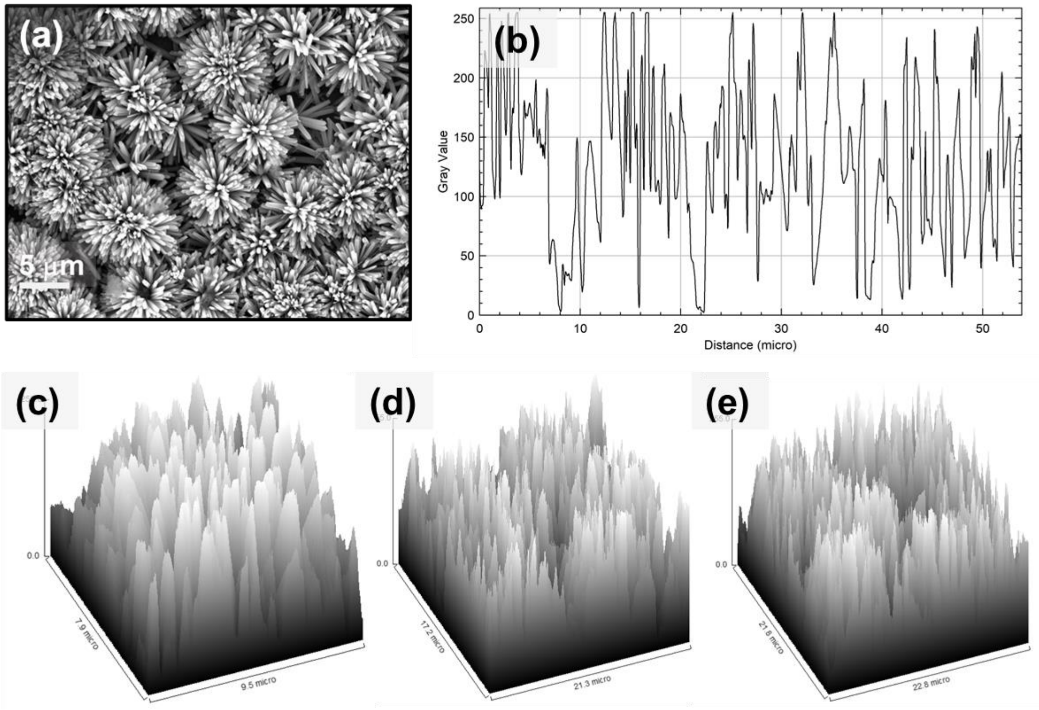

Fig. S1. (a) Plain-view FESEM image of GTNs. (b) Schematic representation of the FESEM plot profile, (c-e) different surface area plot analysis (ImageJ Software) of GTNs.

Quantitative image analysis (ImageJ software) was employed to estimate surface area by incorporating surface roughness and morphological features. Extract line profiles from the FESEM images to calculate roughness values along specific lines [60]. Surface roughness is usually characterized by one of the two statistical height descriptors advocated by the American National Standards Institute (ANSI) and the International Standardization Organization (ISO) [61].

For a digitized profile of length with heights z_i, i = 1 to N,

where N represents the number of measurements, average height parameters are given as

$$R_a = \frac{1}{N}\sum_{i=1}^{N}|zi - \bar{z}| \qquad (1)$$

zi is the height at each data point.

$\bar{z}$ is the mean height over the profile length [62]. A center-line is defined as the line equidistant from the profile such that the area above and below it is equal. Ra, CLA (center-line average) represents the arithmetic mean of the absolute vertical deviations from this center-line.

Figure S1 a) presents the schematic representation of the FESEM of GTNs was used to analyse Specific Surface Area (SSA) [21]. Figure S1 b) represents the FESEM plot profile to calculate the roughness

factor (Ra). Figure S1 (c-e) shows different surface area plots were taken to determine the Ra, to calculate the SSA of GTNs. The specific surface area of GTNs considering surface roughness, can be determined using the following equations.

Calculate the geometric surface area for a square thin film with side length L:

$$A_{projected} = 2\times L^2 \qquad (2)$$

Adjust for Surface Roughness

$$A_{actual}=A_{projected} \times R \qquad (3)$$

Calculate Volume and Mass:

$$\text{Volume (V)} = L^2\times T \qquad (4)$$

$$\text{Mass (m)} = \text{Density } (\varrho)\times\text{Volume (V)} \qquad (5)$$

Specific Surface Area

$$SSA = A_{actual}/m = \frac{2\times L^2\times R}{\rho\times L^2\times T} \qquad (6)$$

Calculations were done by using the given parameters of GTNs,

Sample length (L) = 2 cm

Thickness (T) = 8.4 μm = 0.00084 cm (since 1 μm = 0.0001 cm)

Density (ϱ) = 4.13 g/cm^3

Roughness factor (R) = 32 (R was Obtained from Figure S1. (a-e))

Substitute the values in equation (2)

$$SSA= \frac{4\ cm^2\times 32}{4.13\ \frac{g}{cm^3}\times 2^2\times 0.00084\ cm^3}$$

$$= \frac{128\ cm^2}{0.013876\ g}$$

$$SSA = 9211.5\ cm^2/g = 0.921\ m^2/g.$$

References:

60. Smith. M.F, Limwichean S, Horprathum. M, Chaiprapa. J, Aye. W.W. Chananonnawathorn, C.b Patthanasettakul, V. Eiamchai, P. Nuntawong, N. Klamchuen. A, Thin Solid Films 2022, *751*.

61. Bhushan. B, Mod. Tribol. Handb. Vol. One Princ. Tribol, **2000**, 49–119.

62. Sliz. R, Eneh. C, Suzuki. Y, Czajkowski. J, Fabritius. T, Kathirgamanathan. P, Nathan. A, Myllyla. R, Jabbour. G, RSC Adv, **2015**, *5*, 12409–12415.

21. Hameed. N.A, Ali. I.M, Hassun. H.K, Energy Procedia **2019**, **157**, 84–89.
